# Better governance starts with better words: why responsible human tissue research demands a change of language

**DOI:** 10.1186/s12910-022-00823-7

**Published:** 2022-09-01

**Authors:** Michael A. Lensink, Karin R. Jongsma, Sarah N. Boers, Annelien L. Bredenoord

**Affiliations:** Department of Medical Humanities, University Medical Center Utrecht, Utrecht University, PO Box 85500, 3508 GA Utrecht, The Netherlands

**Keywords:** Governance, Biobanking, Ethics, Framing, Human tissues

## Abstract

The rise of precision medicine has led to an unprecedented focus on human biological material in biomedical research. In addition, rapid advances in stem cell technology, regenerative medicine and synthetic biology are leading to more complex human tissue structures and new applications with tremendous potential for medicine. While promising, these developments also raise several ethical and practical challenges which have been the subject of extensive academic debate. These debates have led to increasing calls for longitudinal governance arrangements between tissue providers and biobanks that go beyond the initial moment of obtaining consent, such as closer involvement of tissue providers in what happens to their tissue, and more active participatory approaches to the governance of biobanks. However, in spite of these calls, such measures are being adopted slowly in practice, and there remains a strong tendency to focus on the consent procedure as the tool for addressing the ethical challenges of contemporary biobanking. In this paper, we argue that one of the barriers to this transition is the dominant language pervading the field of human tissue research, in which the provision of tissue is phrased as a ‘donation’ or ‘gift’, and tissue providers are referred to as ‘donors’. Because of the performative qualities of language, the effect of using ‘donation’ and ‘donor’ shapes a professional culture in which biobank participants are perceived as passive providers of tissue free from further considerations or entitlements. This hampers the kind of participatory approaches to governance that are deemed necessary to adequately address the ethical challenges currently faced in human tissue research. Rather than reinforcing this idea through language, we need to pave the way for the kind of participatory approaches to governance that are being extensively argued for by starting with the appropriate terminology.

## Background

Modern healthcare is increasingly moving towards precision medicine: the idea that by targeting treatment using individual patient characteristics, the risk–benefit ratios and cost-effectiveness of therapies can be improved [3, 4]. The rise of precision medicine has led to an unprecedented focus on the storage and use of human biological material and the data derived from these samples via biobanks [5, 6]. Moreover, rapid advances in stem cell technology, regenerative medicine and synthetic biology are leading to more complex human tissue structures and promising new applications, such as ‘organoid medicine’, stem cell-based drug discovery, or clinical gene-editing [7–11]. The ethical challenges of storing and using human tissue for research or clinical purposes have been the subject of extensive academic debate [12–16]. These debates have led to increasing calls for longitudinal governance arrangements between tissue providers and biobanks that go beyond the initial moment of obtaining consent, such as closer involvement of tissue providers in what happens to their tissue, and more active participatory approaches to the governance of biobanks. However, in spite of these calls, such measures are being adopted slowly in practice, and there remains a strong tendency to focus on the consent procedure as the tool for addressing the ethical challenges of contemporary biobanking [1, 2, 17–19].

Both in biobank practice and academia, the provision of tissue is phrased as a ‘donation’ or ‘gift’, and tissue providers are referred to as ‘donors’ [20–26]. In this paper, we argue that this language pervading the field of human tissue research is one of the reasons why there remains such a strong focus on informed consent in governance frameworks for biobanks, in spite of the extensive calls for more participatory approaches to governance measures that go beyond the consent procedure. In the medical context, the notion of a donation expresses the underlying assumption that people are owed nothing or at least very little in return for providing their tissue to research. We will show that this is not just a semantic issue, but poses a substantial ethical problem. This is because language is performative, meaning that the words that are used in practice affect the norms and values that exist within that (professional) sphere, and in turn shape ideas about what is ethically sound. The effect of using ‘donation’ and ‘donor’ therefore facilitates a professional culture in which the nature of this act is perceived as an altruistically motivated surrender of control that is ethically justified by obtaining valid informed consent. Framing biobank participants as passive providers of tissue free from further considerations or entitlements therefore hampers the kind of participatory approaches to governance that are deemed necessary to adequately address the ethical challenges currently faced in human tissue research. It is necessary to start using terminology that promotes the kind of participatory approaches to governance that are being extensively argued for.

## More than semantics

Our argument starts from the claim that we must change the dominant language used in human tissue research to promote ethically sound professional norms and values. That such a change is not a matter of mere semantics stems from the well-established fact that language possesses *performative* qualities when used in practice, meaning that it affects power dynamics and the formation of identities, and shapes the roles and responsibilities that govern a professional field [27–31]. A well-known example of the performative power of language is *framing*, which means that specific names or words highlighting (or obscuring) certain characteristics of that to which they refer. In this process, they shape our experience of and the meaning we attribute to those phenomena [32]. As has been argued before within the context of biobanking, language plays a performative role in setting the ethical stage by providing a frame for the types of questions and challenges that are considered legitimate within biobanking [33, 34].

The importance of using the appropriate language in the domain of medical research has historical precedence: in 1998, the British Medical Journal promptly changed its editorial policy so that the word ‘subject’—which was argued to signify ‘subservience’—would no longer appear in article abstracts. Instead, people participating in medical trials would be referred to as ‘participants’, to echo their interests in being more closely involved in the design and conduct of research [35]. Similarly, our calls for more appropriate language in tissue research are more than merely a matter of semantics, as this would by extension then also apply to the move from subject to participant that is now well-established [36].

## The dominant language in biobanking frames the provision of tissue as an altruistic act that justifies surrender of control

So what explains the discrepancy between the need for more participatory approaches to governance in biobanking, and the emphasis on the consent procedure almost as an ethical panacea? We contend that one of the reasons for the persistence of this idea are the performative effects of the language of “donation” that pervades the field of research biobanking.

Historically, the notions of gift-giving or donation have become standardized in the medical context since the publication of Richard Titmuss’ influential work *The Gift Relationship* (1970). Titmuss was concerned that certain areas of social life he considered important for social cohesion—such as the ‘right to altruistic expression towards others’—were threatened to be commercialized via the commodification of human biological material for research [37]. To neutralize this threat, Titmuss proposed that the provision of human biological material (in his case, blood) for research should be conceptualized as a *gift*, since gifting constitutes an altruistic act free of *any* reciprocal expectations [33]. The gift-metaphor helped shape current professional culture, in which tissue provision is viewed as a *donation* and those providing tissue as *donors.* Titmuss emphasized the altruistic nature of providing tissue as a form of solidarity with the sake of research, and sought a way to justify the ‘surrender of control’ over how they are used [25, 38].

The notion of ‘donation’ is intricately connected to the notion of ‘gifting’. In the US, a donation is legally defined as ‘the act by which the owner of a thing voluntarily transfers the title and possession of the same from himself to another person, without any consideration; a gift’ [39]. Similarly, as observed by Tutton (2004), the British Medical Research Council deliberately refers to tissue provision as a donation, because the idea of gift-giving facilitates research by emphasizing its ‘altruistic, non-reciprocal’ nature [25]. Other conceptualizations of ‘donating’ exist, and some of these definitions do include a notion of gift-giving with a reciprocal or relational element [40]. However, while she argues that donation (of medical data) *should* imply ‘relationality’ and ‘indirect reciprocity’, the point of departure for her argument is that the (legal) definition of a donation wrongfully suggests otherwise. While she makes an important point that actually resembles ours, it also underlines the kind of connotation that the notion of donation apparently has, thus proving our point: in the context of biobanking, it is the Titmussian conceptualization of a donation as an altruistic act that justifies a surrender of control that is still tremendously influential. This can also be derived from the extensive criticism specifically targeting this idea: many have argued that such a conceptualization of this act caters to the interests of those seeking to use the tissues, which likely *explains* its dominance, but has also led to substantial and growing concerns about exploitation and rights violations [20, 21, 24, 38, 41–45].

The point is that the meaning conveyed by the notion of donating tissue is at odds with the calls for increased control and continuous involvement of tissue providers. While these calls are becoming more urgent, the dominant language to describe this act is rooted in the idea that providing tissue to a biobank rather constitutes an altruistically motivated surrender of control, free from consideration or any (reciprocal) expectations. Regardless of whether the act of providing tissue to research should be conceived or defined as reciprocal, at the very least tissue providers are entitled to certain obligations from tissue users towards the protection of their rights and respect for their interests. Titmuss’ emphasis on the need to adopt the gift-metaphor to conceptualize the relationship between tissue providers and users is a testimony to the importance of using the right words or phrasing in this respect.

## The ethical challenges of contemporary research involving human biological material demand ongoing involvement and control

Contemporary human tissue biobanking takes place in a context of rapid advances in fields such as stem cell research, regenerative medicine, synthetic biology and genetics, and applications for personal clinical benefit [7, 46–48]. These developments both stress the urgency of re-evaluating already known challenges and also present us with novel ethical questions [49]. There are several reasons why the meaning that is being conveyed through the language of ‘donation’ and ‘donor’ as defined above is inappropriate in light of the ethical challenges of contemporary biobanking.

First, biobanks generally store human tissues and data for long term use. Specific details about future use of tissues are therefore unknown at the time consent is given. This means that biobank participants do not have influence/knowledge whether their samples and data will be used according to their values and preferences [50]. While this inherent limitation of consent in biobanking has been extensively discussed, the rapid pace at which advances in tissue technology are leading to more complex and ethically sensitive applications underline the urgency of addressing this issue. Emerging complex tissue technologies, such as brain organoids or synthetic embryos, have given rise to specific ethical questions concerning the moral status of such novel entities [49, 51]. Moreover, applications such as chimaera research, ‘brain emulation’ with cerebral organoids, or in vitro gametogenesis have sparked discussion about ethical boundaries [10, 51–53]. Lastly, the application of increasingly complex parts of human bodies as marketable commodities also raises questions about bodily integrity, conceptualizations of the self, and human dignity [47, 54, 55]. Crucially, empirical studies demonstrate that both researchers and tissue providers hold different personal values and opinions about these topics [26, 56–59]. Moreover, the fact that such complex ethically sensitive tissue products can be cultivated from ordinary samples—such as skin cells or blood—broadens the scope of these considerations to include people who have already provided samples in the past. These developments have led to extensive calls for more longitudinal control and involvement over how tissue is being used that go beyond initial consent [60, 61].

Second, the combination of rapid biotechnological developments and the rise of precision medicine have led to an unprecedented economic interest in human biological material, resulting in strong profit-driven incentives for commercial entities to further push the use of tissue and data collections stored in biobanks [38, 46, 62–65]. Crucially, biobanks themselves are often dependent on some form of profit-generation to be feasible; involvement of industry is thus an important factor for realizing the potential (clinical) benefits of research [66–68]. At the same time, there is evidence that commercial interests may affect the availability of samples, such as via Material Transfer Agreements to restrict access [65]. Moreover, as the economic potential of human biological material and data increases, so does the incentive for tissue users to capitalize on this potential, which could put pressure on ethical conditions such as privacy or the right to withdraw [6, 64, 69, 70]. We know from empirical research that tissue providers feel ambivalent about commercial involvement: while the importance of involving industry is often acknowledged, many are also concerned about the dominance of profit-driven interests and exploitation [57, 64, 71]. These are legitimate reasons to provide biobank participants with more ongoing control and ownership over their biological material.

Third, precision medicine tissue research blurs the boundary between (commercially driven) laboratory work and clinical patient care, which has ethical implications. Whereas doctors are legally charged with the responsibility to act in the interests of their patients, there is no consensus on whether and to what extent these responsibilities apply to tissue users [7, 72–78]. For example, there have been calls for making the disclosure of general and individual results an ethical duty for stem cell and genomics researchers [79–81]. This is crucial in the clinical context, in which the persons provided tissue are often dependent for their own health on how their samples are used by other parties, and the tissue may have been provided with the expectation that it be used in ways that benefit the person or the patient community as a whole [57, 67]. However, tissue use may be prioritized based on which types of applications are most profitable, or best satisfy researchers’ academic aspirations [70, 80]. The existence of different, sometimes competing interests between tissue providers and tissue users stresses the need for ongoing ‘benefit sharing’ arrangements, in which the interests of patients providing tissue are represented fairly alongside the interests of other stakeholders, and allow them to make their interests known [2, 54, 69, 72, 82, 83].

## The appropriateness of consent relies on sound governance rather than being its foundation

The challenges of modern (complex) tissue biobanking thus increasingly underline the importance of providing tissue providers with more longitudinal ownership and control over how their tissue and data are being used [63, 65, 84–88]. At the same time, there has been and still exists a strong emphasis on different consent models as the go-to approach for addressing the ethical challenges in tissue research [1, 89–93]. The ethical requirement of obtaining voluntary, informed consent serves a crucial moral purpose in biobanking, namely protection against violations of autonomy and bodily integrity and preventing illicit procurement of tissue [13, 18, 19]. But consenting to participate in a specific trial is a fundamentally different kind of decision than permitting an enterprise or an institution to govern one’s bodily material or data for unspecified purposes and duration [94–97]. The real locus of concerns and interests for tissue providers is situated in more ongoing forms of participation, control, and involvement. This is not only demonstrated by the nature of the challenges of contemporary biobanking described earlier; several recent empirical studies show that tissue providers wish to have a say in how research results are disclosed to them, to have more insights into the purposes for which their tissue is being used and what their contribution has led to, and to be more closely involved in the governance of biobanks [57, 93, 98–101].

The continuous nature of these interests stresses the urgency of more *ongoing* governance measures *in addition to* the ex ante consent procedure. In fact, the 2016 revision of the guidelines for research involving humans developed by the Council for International Organizations of Medical Sciences and the World Health Organization underlines this point, stating that ‘the ethical acceptability of broad informed consent relies on proper governance’. According to the report, this includes participatory arrangements, and sound policies for communication of unsolicited findings, research outcomes, benefit-sharing, and prevention of adverse effects on tissue providers’ rights and welfare [102, 103]. The point is that addressing these challenges does not demand “deep” or “dynamic” consent or any other kind of consent, but rather demands sound, participatory governance structures to be in place.

It may be true that most human biological material used in research is not provided through active choice, and that when it is, it was provided based on informed, voluntary consent. But the fact that people accept the conditions currently offered to them for the sake of research or health benefits is insufficient moral justification for the lack of *ongoing* respect for the rights, interests, and values of tissue providers in the conditions that are presented to them. On the contrary: the high level of trust that people have in the research enterprise and their willingness to participate rather underlines the need to ensure that these interests are taken sufficiently into account [104]. Both participatory arrangements and the consent procedure are crucial elements of any biobanking governance framework, but they have distinct moral purposes: where participatory governance puts the importance of a continuous, bi-directional relationship between tissue users and tissue providers at the center, the purpose of the consent procedure is to ensure that the decision to transfer one’s bodily material to a biobank is made voluntarily and well-informed. In other words, it makes little sense therefore to solve challenges in biobanking related to the lack of involvement, ownership of tissues and control over how they are used by focusing on the consent procedure. It is perhaps an indication of this mismatch between problem and solution that explains the lack of consensus that still characterizes the debate about consent [105]. As Susan Cargill states, we need to stop trying to ‘fit the square peg of biobanking permission into the round hole of research informed consent’ and rather explore alternatives [94].

## The fixation on consent and the language of ‘donation’ are mutually reinforcing

So far, we have separately criticized the fixation on the consent procedure as the core ethical condition in biobanking governance, and the pervasive use of the notion of donation. The importance of using appropriate language becomes especially clear with the observation that there is a mutually reinforcing dynamic at play between these two phenomena. On the one hand, the emphasis on voluntary, informed choice as the core ethical condition for the provision of tissue suggests that the consent procedure constitutes most of what is ethically *needed.* On the other hand, by using language in which the act of providing tissue is conceptualized as non-reciprocal (i.e., a ‘donation’), its performative qualities set the bar in terms of what tissue providers are ethically *owed.* Through the process of framing, such language thus shapes the professional and ethical norm that a given consent constitutes sufficient moral justification for cutting tissue providers off from ongoing involvement and control over the use of their tissue [45, 46, 63]. And the idea that informed choice is what matters most to tissue providers works towards justifying the assumption that it is ethically appropriate to position them as passive participants, thus legitimating the use of words like ‘donation’ and ‘donor’. In fact, the consent procedure may be understood as facilitating the ‘disentanglement’ between person and tissue, by which the latter is changed into an exchangeable product with market value [34, 47, 50]. By giving their consent, tissue providers effectively agree to give up further rights, entitlements or control pertaining to their tissue, which provides third parties more flexibility and freedom in the distribution and application of samples in ways that cater mostly towards their own interests [54, 65]. In an empirical study, we observed a tendency among professionals involved in organoid research to emphasize the instrumental value of the consent procedure as an administrative tool [58]. Informed consent in this way functions as an ethical straw man: a checkbox that serves to prepare bodily material for use without further obligations rather than protect the rights and respect the interests of tissue providers.

The kind of participatory governance that is necessary, in our view, starts from the assumption that people are not only entitled to the protection of their legal rights, but also that arrangements should be in place that envision a bi-directional relationship between tissue providers and tissue users, in which tissue providers are empowered with more ongoing involvement with what happens to their tissue, and control over the conditions that dictate how their bodily material is governed. Crucially, participatory governance is not a one-size-fits-all affair: it should also allow people to have a say in the degree of involvement or control they wish to have, to accommodate for the different needs and preferences that exist between individuals [70]. As long as the dominant language to describe tissue providers frames them as ‘donors’, the foundation (i.e., professional norms and values) from which to realize a change towards this kind of participatory governance will remain weak. This is particularly problematic in the context of precision medicine, where the prospect of potential clinical benefits particularly motivates patients to provide their tissue, even under sub-optimal conditions. If anything, these are reasons to put *additional* efforts into ensuring that governance adequately respects the interests of tissue providers [12, 41, 106, 107]. Moreover, as it stands, tissue providers generally trust that research institutions will use their tissues responsibly [20, 57, 65, 71, 101]. But as Dickenson (2008) observes, framing people’s provision of tissue as a gift or donation while companies and researchers are treating them as economically valuable can ‘provoke in donors a sense of being duped’, which may erode their trust [47]. The language of ‘donation’ that currently still pervades the field of human tissue biobanking is therefore not only misaligned with the interests of tissue providers, but its performative effects on the formation of professional norms and values also resists improvement or change towards ethically sound governance frameworks.

## Better governance through better words

Consistent use of the language of ‘gifting’, ‘donating’ and ‘donors’ thus shapes and maintains the idea in both professional as well as civil spheres that providing tissue to a biobank should be seen as a an altruistically motivated act, in which control is surrendered free from expectations in return. In this way, it effectively hampers the development and implementation of governance frameworks that are better able to tackle the challenges and risks involved in tissue research. Moreover, as demonstrated by the many empirical studies cited in this paper, even though people may refer to themselves as ‘donors’ and to the provision of their tissue as a ‘gift’, they simultaneously have expectations and preferences about how their bodily materials are being governed and how their interests are being protected. A qualitative study specifically aimed at investigating how providers of biological material perceive the notion of donation was published by Locock and Boylan in 2014. The study observed that people’s attitudes to what it means and implies are ambiguous, and that there is a tendency to view a donation as a ‘gift free of all claims’ [21]. Another study by Dixon-Woods et al. observed that some people were offended by the language of gifting, because of “its status as a 'marketing ploy' and potential for putting pressure on people to consent” [88]. With regards to professionals, Richard Tutton observed that ethical review boards, medical councils, and researchers conceptualize the donation of body tissues as a one-way transaction by using the language of ‘donation’ and ‘gift relationships’ [25, 108]. Lastly, Rhonda Shaw observes that how people experience a ‘donation’ depends on the kind of tissue, and to whom it is being provided [24, 109]. We know that tissue providers are interested in increased control over how their tissue is being used, and desire ways of being more continuously involved, particularly when sensitive tissue or applications are concerned or when patients are involved [59, 110, 111]. The existence of such interests and expectations among people who provide their bodily material to research is difficult to reconcile with the use of language that normalizes the idea that the act of providing tissue should be seen as an altruistically motivated surrender of control. On the contrary: people are deserving of respect for their needs and interests in return for providing their tissue, and the language we use to describe them should echo this view.

One frequently used response to calls for more control and involvement of tissue providers in governance is that enforcing such obligations stifles the practical and economic viability of the research environment [7, 37, 112]. This is indeed an important concern. However, we believe it has insufficient merit, since there is little reason to assume that promoting closer continuous involvement of biobank participants (through language or otherwise) will pose a threat to the feasibility of the research enterprise. Many such governance solutions have already been proposed with convincing arguments for technical and financial viability, thanks to improved digital possibilities such as ongoing communication about research activities and results, closer involvement at the level of biobank management and decision-making, real-time (digital) information and control over how tissue is being used, or involving patient organizations in research design and agenda-setting [113–116]. Secondly, there are several legal cases that demonstrate how the lack of obligations from tissue users to tissue providers can lead to costly controversy, poignant legal battles, and human rights violations [111, 117–121]. Rather than stifling tissue research, the implementation of longitudinal, more participatory approaches to governance, in which tissue providers are positioned more like ‘partners’ than as passive sources of research samples, is likely to *increase* its viability by ensuring public trust is maintained [2, 122, 123].

Considering the power of language to shape professional norms and values, we should refrain from using words that assume and suggest passive altruism and a surrender of control. On the contrary, we should strive to use language that works towards normalizing the idea of a more continuous, bi-directional relationship between tissue providers and biobanks, and that facilitates people’s involvement in how their bodily material is governed and used. Fortunately, the fact that language is performative also means that it can be deployed to promote the kind of professional norms and values that will help stimulate the establishment of more participatory approaches to governance [124]. Adopting a language that reflects the considerations mentioned above is crucial, because its performative qualities help promote a *culture* within the research enterprise in which individual tissue providers and the communities they are part of are treated with the ongoing respect and consideration they deserve, and which stimulates people’s sense of having made a meaningful contribution [115, 125].

## Conclusions

Research involving human biological material has become an invaluable component of contemporary healthcare, and emerging technological developments will further increase the value and usage of human tissues and data. To keep being able to reap its benefits, maintaining an economically viable research ecosystem is paramount, and the many aspects of human tissue biobanking that are currently functioning well should not be overlooked. That said, the current status quo in the governance of tissue research has several ethical shortcomings that stem from the lack of involvement and control that people have over how their bodily material is being used. In this paper, we have shown that there is a mutually reinforcing dynamic at play between the central position that is still attributed to informed consent in biobanking governance, and the framing effects of using language pervaded by the notion of donation.

In addition to the lack of attention for the actual interests of tissue providers that is conveyed by the notion of donation, it offers one explanation for why—in spite of the extensive calls for more involvement of biobank participants—biobanking practice has been slow to adopt such participatory approaches to governance frameworks and regulatory standards. Throughout this paper, therefore, we have intentionally refrained from using the notions of ‘donation’ and ‘donor’ except for the purposes of criticism. Instead, we have consistently described them as ‘tissue providers’, ‘participants’, or ‘partners’. Similar to the move away from framing participants in medical research as ‘subjects’, our aim with this strategy was to set an example: we should use the performative qualities of language to promote a much needed transition towards a more involved, continuous relationship between tissue providers and tissue users.

## Data Availability

Not applicable.
